# Low Consistency of Four Brain Connectivity Measures Derived from Intracranial Electrode Measurements

**DOI:** 10.3389/fneur.2014.00272

**Published:** 2014-12-19

**Authors:** Stephen E. Jones, Erik B. Beall, Imad Najm, Ken E. Sakaie, Michael D. Phillips, Myron Zhang, Jorge A. Gonzalez-Martinez

**Affiliations:** ^1^Cleveland Clinic, Cleveland, OH, USA; ^2^Ohio State University College of Medicine, Columbus, OH, USA

**Keywords:** intracranial electrodes, brain stimulation, functional MRI, structural connectivity, functional connectivity

## Abstract

Measures of brain connectivity are currently subject to intense scientific and clinical interest. Multiple measures are available, each with advantages and disadvantages. Here, we study epilepsy patients with intracranial electrodes, and compare four different measures of connectivity. Perhaps the most direct measure derives from intracranial electrodes; however, this is invasive and spatial coverage is incomplete. These electrodes can be actively stimulated to trigger electrophysical responses to provide the first measure of connectivity. A second measure is the recent development of simultaneous BOLD fMRI and intracranial electrode stimulation. The resulting BOLD maps form a measure of effective connectivity. A third measure uses low frequency BOLD fluctuations measured by MRI, with functional connectivity defined as the temporal correlation coefficient between their BOLD waveforms. A fourth measure is structural, derived from diffusion MRI, with connectivity defined as an integrated diffusivity measure along a connecting pathway. This method addresses the difficult requirement to measure connectivity between any two points in the brain, reflecting the relatively arbitrary location of the surgical placement of intracranial electrodes. Using a group of eight epilepsy patients with intracranial electrodes, the connectivity from one method is compared to another method using all paired data points that are in common, yielding an overall correlation coefficient. This method is performed for all six paired-comparisons between the four methods. While these show statistically significant correlations, the magnitudes of the correlation are relatively modest (*r*^2^ between 0.20 and 0.001). In summary, there are many pairs of points in the brain that correlate well using one measure yet correlate poorly using another measure. These experimental findings present a complicated picture regarding the measure or meaning of brain connectivity.

## Introduction

The importance of brain connectivity is self-evident given the underlying network structure of the brain. Structural MRI, which interrogates each point in the brain, is invaluable to science and medicine. The point-to-point relationships of connectivity imaging are equally as invaluable, if not more so, given the inherent network nature of brain functions. As such, connectivity imaging represents the next step in the continuing evolution of neuroimaging. However, unlike structural imaging, contemporary connectivity analyses have not yielded findings of relevance for treatment in individual patients.

There are many measures of connectivity, which could be dichotomized into functional and structural. Examples of structural connectivity are derived from invasive measures such as axonal tracing or nuclear tracing, with the prime non-invasive method of MRI using diffusion-weighted imaging (dMRI). Examples of functional connectivity include measures derived from scalp EEG, intracranial EEG, PET studies, cortical thickness studies, task-related fMRI, and resting state fMRI (rsfMRI). All these methods have advantages and disadvantages, related to spatial and temporal resolution, coverage, effectiveness, and invasiveness. Given numerous methodologies and metrics of connectivity (1, 2), it is natural to compare them with the hypothesis that if the metrics are sensitive to the underlying network architecture of the brain, then the connectivity measures should strongly correlate with each other. This inquiry raises the question about the exact definition of connectivity, and what could be considered the “gold standard.”

The paper focuses on recent measurements obtained from a group of eight patients with medically intractable epilepsy, who underwent both invasive electroencephalographic (EEG) and evoked potentials mapping with implantable intracranial electrodes and advanced neuroimaging with MRI. Using these methods, a total of four modalities of connectivity were explored: structural connectivity using dMRI, functional connectivity using rsfMRI, functional connectivity using precise electrical stimulation and recording (cortico-cortical evoked potentials, CCEPs) from intracranial electrodes, and combined intracranial stimulation and BOLD fMRI. These four modalities of connectivity are compared on a pairwise basis, and we show that although the comparisons reveal statistically significant correlations, the correlation values are modest. Furthermore, these methods, as commonly interpreted today, do not reach the same consensus. In other words, there are many pairs of points in the brain that correlate strongly using modality A, but correlate weakly using modality B, and visa versa. A possible future method to unify these differences may incorporate a mathematical model of brain function, which would permit the translation of one connectivity measure to another.

## Materials and Methods

Four different measures of connectivity are used in this study; all measured in epilepsy patients who underwent an invasive evaluation to better localize and map the extent of the epileptogenic zone (EZ). The four measures are functional connectivity derived from electrophysiological response to electrical stimulation (CCEPs); functional connectivity derived from low-frequency BOLD oscillations in the rsfMRI; functional connectivity derived from simultaneous direct electrical stimulation and BOLD functional MRI (DES-fMRI); and structural connectivity derived from dMRI using high-angular resolution diffusion imaging (HARDI).

After obtaining IRB approval, a total of eight patients were enrolled. All patients were recruited from the Cleveland Clinic Epilepsy Center with a diagnosis of intractable focal epilepsy, and underwent an intensive evaluation culminating in the placement of intracranial electrodes. All patients underwent CCEP stimulation, with multiple locations of stimulation, including the Broca’s speech region, and the presumed EZ. The first four patients had HARDI and rsfMRI performed prior to implantation, and the last four had HARDI and rsfMRI performed after implantation. These measurements were performed as a “piggy back” during their standard clinical care, and did not interfere with their clinical care. Table 1 lists some clinical characteristics of the patients and the different modalities that were measured.

**Table 1:** **Details of intracranial electrodes placed in eight patients**.

Patient Number	Age	Implantation	Number of intracranial contacts	Number of CCEP stimulations	dMRI rsfMRI	DES-fMRI	Location of stimulation
1	45	SDG^a^	123	4	Y		Left Broca, Left Wernike, Ictal onset zone ×2
2	40	SEEG^b^	57	9	Y		Multiple bi-occipital and right temporal
3	19	SDG	106	7	Y		Left Broca, Wernike, ictal onset zone ×3
4	40	SEEG	104	5	Y		Multiple right frontoparietal
5	25	SEEG	120	1		Y	Left orbito-frontal
6	41	SEEG	130	1		Y	Right posterior cingulate
7	42	SEEG	160	1		Y	Right peri-insula
8	54	SEEG	130	1		Y	Right orbito-frontal

*^a^SDG: subdural grids*.

*^b^SEEG: stereoencephalography*.

### Cortico-cortical evoked potentials

All CCEP stimulations used a GRASS used current-controlled Grass Technologies S88 and SUI-7 units (Astro-Med), with the following parameters: 1 Hz unipolar pulses with alternating polarity between pulses, 0.3 ms pulsewidth, with variable current (4–15 mA), applied across an adjacent electrode pair. An optical current isolator was used to ensure that the patient was isolated from ground. For each CCEP stimulation location shown in Table 1, between 16 and 60 (typically around 45) stimulating pulses were sent from the chosen electrode pair, and waveform responses were recorded from all other implanted contacts, at a 1 kHz recording rate. Four patients also had CCEP performed while simultaneously undergoing MRI, and for these patients a stimulation frequency of at least 10 Hz was required to elicit a robust BOLD response (3). The CCEP voltage waveforms obtained at each electrode were averaged, discarding any outliers that were usually due to motion and other artifacts. Because of the alternating polarity of each pulse, most of the stimulus artifact was removed, and average waveforms could be reliable seen as early as 5 ms after the stimulus. A scalar baseline was subtracted from each waveform, derived from an average of the waveform during a 40 ms window obtained just before the stimulus. There are many methods to “score” the strength of the averaged CCEP waveform, which can be used as the measure of electrophysiological connectivity. Typically, these methods use the average voltage during a time window after stimulus. Other methods can use the slope, latency, integral, magnitude, and other features of the voltage waveform. For this paper, the chosen time window depended on the other modality used to compare CCEP connectivity: for comparison with structural connectivity, a 5 ms window starting 10 ms after the stimulus was used to reflect the rapid first-pass excitation of distal cortex; for comparison with resting state connectivity a 100 ms window starting at 20 ms was used to reflect the longer timescale of an integrative process.

### Resting state fMRI

For all studies, rsfMRI was performed on a 3T Siemens Trio (Siemens Medical Solutions, Erlangen, Germany), using a whole-brain EPI sequence: 132 repetitions of 31 4-mm thick axial slices; TE/TR, 29 ms/2,800 ms; matrix, 128 × 128; FOV, 256 mm × 256 mm; receive bandwidth, 250 kHz. EPI data were unwarped using a field map prior to coregistration to the unwarped diffusion acquisition space (described below in Section Structural connectivity), which was used to cross-compare the different measures. Patients were instructed to rest with their eyes closed and refrain from any voluntary motion. The data were corrected for motion, adaptive physiologic noise sources (4, 5), and second-order motion (6). The data were interpolated to the DWI space (see below). Functional connectivity maps of the brain were produced using a seed approach, yielding a pairwise temporal correlation coefficient to every other brain voxel. The seed was selected to be the site of stimulation. The time waveforms used in the correlation were the average waveforms of the 27 voxels of a voxel and its nearest neighbors, excluding any CSF-containing voxels.

### Direct electrical stimulation and functional MRI

Direct electrical stimulation and functional MRI (DES-fMRI) is a recently developed modality (3) in which simultaneous fMRI is acquired during stimulation of a single intracranial electrode. The procedure was conducted in an intraoperative MRI suite with the patients under general anesthesia. Using appropriate stimulation frequencies and currents (typically around 20 Hz and 4–8 mA), robust BOLD activation could be generated both proximal and distal to the electrodes. Typically, the activation occurred in patterns that reflect the underlying network. Both positive and “negative” activation could be triggered. Activation could also be induced by white matter stimulation. Using this method, the connectivity metric between the stimulation point S and another point P can be defined as the degree of BOLD change or its statistical significance at point P. Thus, the usual 3D BOLD maps can be viewed as a connectivity map. Prior to DES-fMRI the patients had a comparable CCEP stimulation performed outside of the MRI with all electrodes in place and recording the stimulation’s response, thereby measuring electrophysiological connectivity. By co-localizing the electrode location to the DES-fMRI, a comparison could be directly made between electrophysiological connectivity and DES-fMRI connectivity.

### Structural connectivity

High-angular resolution diffusion imaging images were obtained on a Siemens Trio (Siemens Medical Solutions, Erlangen, Germany) with a standard 12-channel head coil. The HARDI acquisition provided whole-brain coverage with 2.5 mm isotropic voxels (256 mm × 256 mm FOV, 102 × 102 matrix, 48 slices. TE = 77 ms, TR = 6500 ms, BW = 1442 Hz/pixel, partial Fourier factor = 5/8, 61 non-collinear diffusion-weighting gradients with robust ordering with *b* = 1000 s/mm^2^ and 7 *b* = 0 volumes, two averages). Warping effects were addressed by using static image-based unwarping (7) on the diffusion data prior to diffusivity calculation. Motion correction was performed with an iterative algorithm (8) that updated gradient vectors (9). Fiber orientation distributions were calculated in each voxel by spherical deconvolution (10) with user-independent optimized regularization (11). Local transition probabilities were calculated by integrating over the solid angle of a vector connecting each voxel with its 26 neighbors.

The cross-modal comparison performed in this project places additional demands on metrics to measure structural connectivity. For example, since intracranial electrode contacts can be placed in any arbitrary place in the brain, the task of comparing structural to electrophysiological connectivity requires that the structural metric be able to assess or “score” a connection between any two points in the brain. Since these two points may not necessarily both lie along a large fiber track, deterministic methods will fail and probabilistic methods are favored. A further demand is that the “seed” and/or “target” points may lie on the cortical surface, in a region of low FA that hinders the reliable start to a trajectory. One difficulty with probabilistic methods is that they can be computationally inefficient, if they adopt a method of randomly forming a path and discarding it if it fails to reach a target. To account for this difficulty a partial differential equation (PDE) approach was developed that is the solution of a probabilistic method assuming an infinite number of trials, akin to the underlying relationship between a PDE and Monte Carlo solution to classes of differential equations (12). Details of this method are presented in the Data Sheet 1 in Supplementary Material.

### Coregistration

The processes of coregistration of the electrode positions to the MRI voxels started with a thin section head CT following implantation of electrodes. Then using in-house techniques, all electrode contact positions were identified and recorded (13). The CT scan was then registered to the anatomical scan (T1-weighted MPRAGE) obtained prior to surgery, and the positions of all electrodes translated into the MRI-space. All image registration was affine and performed using FSL FLIRT (14, 15). Segmentation was performed on the T1 MPRAGE images using the Freesurfer package (http://surfer.nmr.mgh.harvard.edu/), whose parcelation maps were interpolated into the dMRI space.

### Paired correlation and statistical measures

For each of the four modalities, as summarized in Table 2, using a single location as a “seed,” a large set of connectivity values can be computed to targets outside of the seed: for the CCEP modality the targets are the other recording electrodes (which can number up to 200), and for each target a connectivity value can be computed; for the other modalities the targets are the remaining voxels occupying cortical gray matter, which can number up to 5000–10,000. For a given patient with an intracranial electrode used for CCEP stimulation, all four measures of connectivity can be computed using the stimulation location as a common seed. This permits a cross-comparison between the modalities; specifically the connectivity from the seed to any target point can be compared between the four modalities. The comparison can be made for all targets in the form of a two-dimensional scatter plot, with each axis representing the magnitude of a modality’s connectivity. One measure of the consistency between two modalities is the Pearson correlation coefficient, such that a value of 1 is a perfect correlation and 0 is no correlation. A *p*-value for the correlation coefficient is also obtained. These values were computed using Interactive Data Language (IDL, Exelis, Boulder CO, USA).

**Table 2:** **Summary of the four measures of connectivity used for comparisons in epilepsy patients who underwent evaluation with intracranial electrodes**.

	Category	Method	Measure of connectivity
1	Electrophysiological-stimulated	CCEP	Mean voltage during short time window
2	Functional-passive	Resting state fMRI	Temporal correlation coefficient, seed-based approach
3	Functional-stimulated	DES-fMRI	*t*-score from a BOLD activation map
4	Structural-passive	dMRI	Product of local connectivities along pathway determined from PDE approach

Aside from AFNI (22), FSL, and Freesurfer routines, all software routines were developed in house using the Interactive Data Language (IDL, Exelis, Boulder CO, USA). All IRB and HIPPA requirements were strictly followed.

## Results

### Cortico-cortical evoked potentials

**Figure 1:**
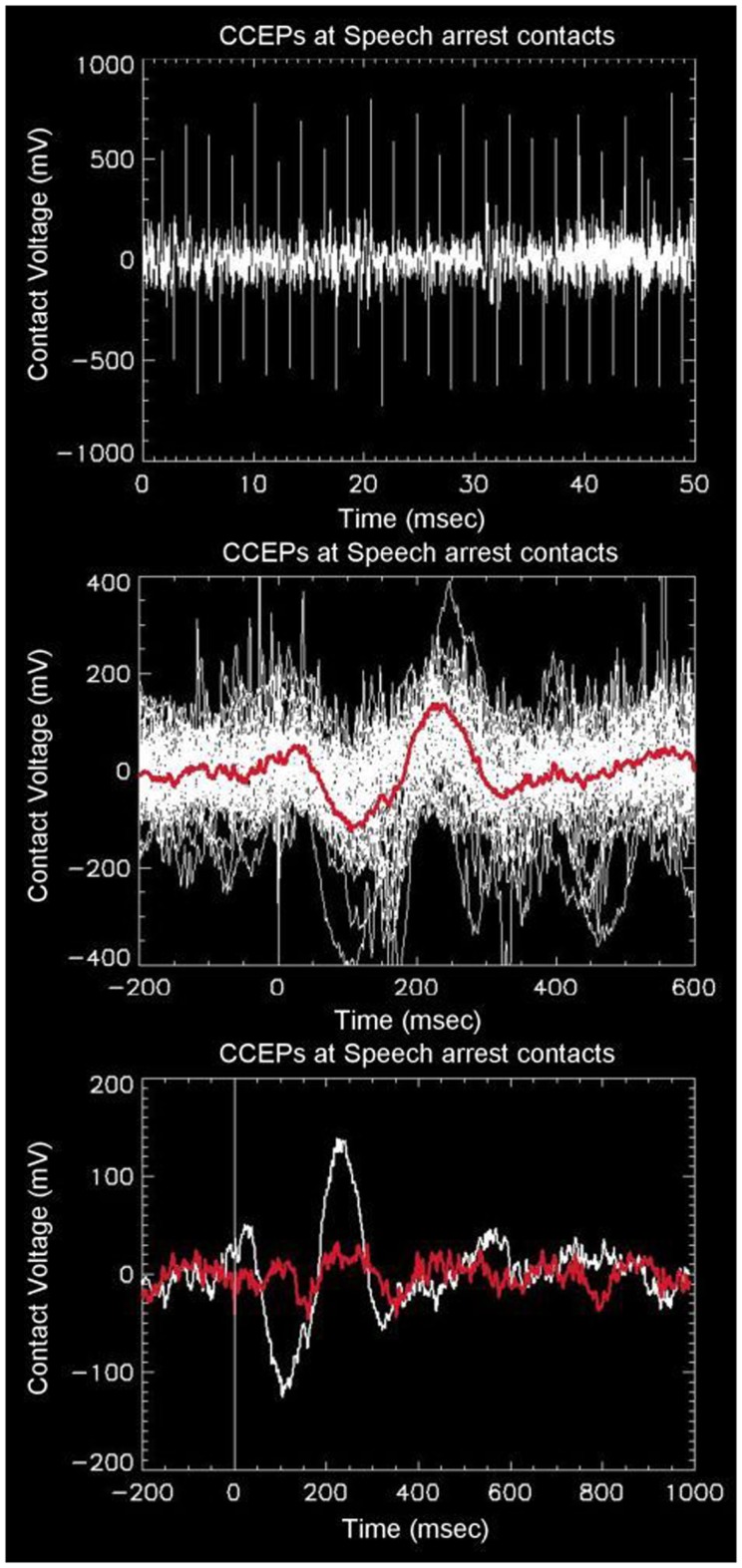
**Top: example of raw data recorded from one intracranial electrode (in this case overlaying the presumed Wernicke’s area in the left angular gyrus) in response to stimulation from left Broca’s region**. There are a total of 46 alternating unipolar stimulations seen as spikes from stimulation artifact. The middle panel shows the overlay of all 46 waveforms as referenced to a common stimulation time. The red line shows the average of the waveforms, with a prominent downward peak at 100 ms, followed by an upward peak at 230 ms. The bottom panel shows the same average waveform overlaid with the waveform obtained from one adjacent electrode 10 mm away, showing strong spatial variability from distal stimulation.

Robust distal and proximal activation is easily elicited with this technique. The top panel of Figure 1 shows a typical example of one complete electrode recording obtained in the parietal lobe of a patient with a large subdural grid array (#1), who was stimulated in the frontal Broca’s regions (as determined earlier by speech arrest using higher currents and frequencies). A series of 46 CCEP recordings are seen via their stimulation artifact (tall alternating spikes), occurring over a recording duration of 50 s. The middle panel of Figure 1 shows an overlay of the 46 CCEP recordings from the same electrode mapped to a common stimulation time point, with the red line showing the mean signal. This plot reveals the degree of variability typical in these electrophysiological experiments. The white line in the bottom panel of Figure 1 shows the mean signal from an electrode in the parietal lobe known to be associated with language (as determined earlier using speech arrest obtained after stimulation at higher currents and frequencies). The observation of speech arrest and the robust CCEP signal implies that both the stimulation and recording points lie on a portion of the language network, i.e., the presumed Broca’s and Wernicke’s area. The overlaid red line was obtained from an adjacent electrode about 10 mm distant, which appears markedly different, showing minimal evoked potential. The difference in these two graphs across 10 mm indicates the spatial scale across which markedly different EP connectivities can be measured using this technique.

**Figure 2:**
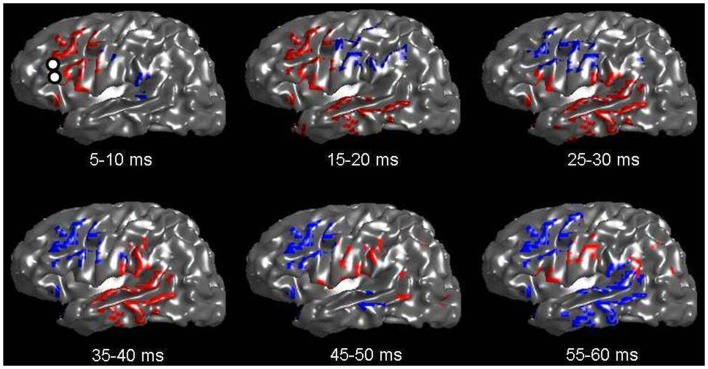
**Voltage patterns recorded from left-sided subdural grids in response to a CCEP stimulation at left Broca’s region (whose electrode-pair is shown as two white circles in the first image)**. The underlay is the inflated brain. Voltages are taken as the average during a 5 ms window. Red represents positive voltages greater than 50 mV; blue represents voltages less than −50 mV. A total of six images are shown with mean voltages taken from the displayed time intervals. Note the rapid evolution of voltage spread, which qualitatively follows the expected connection along the arcuate fasciculus.

Figure 2 shows an inflated surface reconstruction with an overlay of the CCEP response, obtained over a grid array of 120 electrodes. The electrical stimulation was applied to the left inferior frontal gyrus at *t* = 0 ms for a duration of 0.3 ms, at the location shown by the two small white circles in the first image. Red colors represent positive voltage and blue are negative, using a threshold of ± 50 μV, respectively. The displayed time periods are the times during which the voltage was averaged. The figure shows the relatively rapid evolution of the pattern of response during the first 50 ms: a very early negative response is seen around the supramarginal gyrus, followed by intense activation in the temporal lobe which reverses polarity by 50 ms. Other temporal patterns can be seen, and altogether reveals the complex nature of globally evoked potentials stimulated by a point source.

### Resting state fMRI

**Figure 3:**
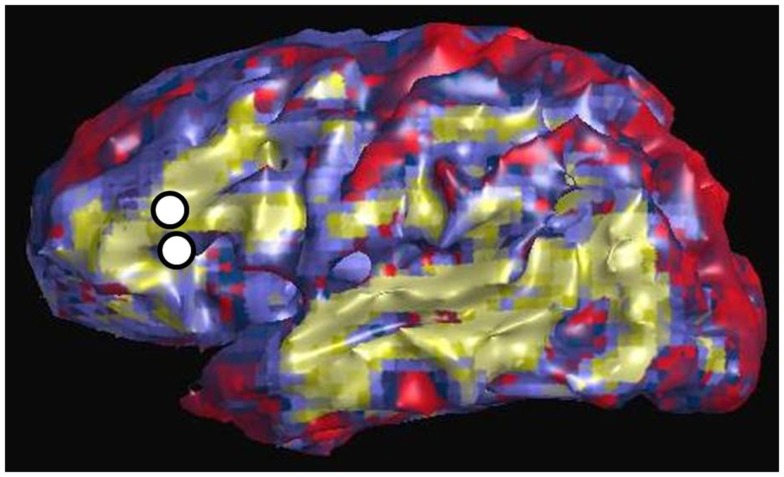
**Resting state connectivity map of left hemisphere, using seed-based approach with seed located at left Broca’s region as indicated by two white circles**. These are the same locations used for CCEP stimulation in Figure 2. The color scale reflects the value of the temporal correlation coefficient, with green-yellow representing any value greater than 0.5. Qualitatively there is strong correlation from Broca’s region to other expected language areas in the left temporal lobe.

Figure 3 shows an example of resting state connectivity as revealed from the temporal correlation coefficients using a seed method. Using the same patient in Figure 2, the seed point was taken as the stimulation location in the inferior frontal lobe, at the functional location of Broca’s area as determined by earlier speech arrest. The green-yellow color indicates a positive correlation value greater than 0.5. The seed points are shown by the white circles, representing the locations of the stimulation electrode pair. The image reveals the widespread network of correlated resting-state fluctuations, which roughly correspond to the presumed distal language regions of the superior and inferior temporal gyri. We define our second measure of connectivity as the magnitude of the correlation coefficient. Due to the inherent three-dimensional nature of MRI, a complete map of connectivity can be produced and compared with other measures.

### Direct electrical stimulation and functional MRI

**Figure 4:**
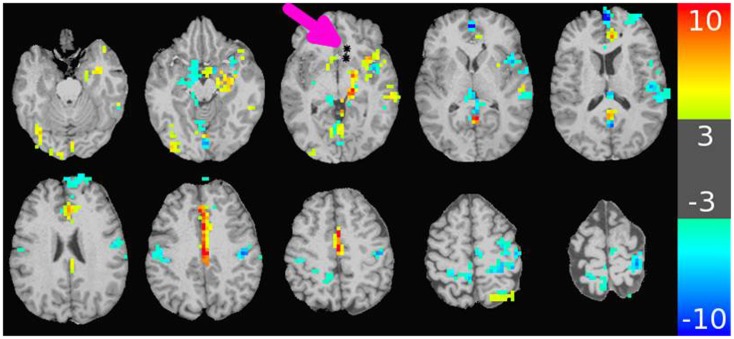
**Map of BOLD response in brain due to stimulation of an electrode-contact pair shown as asterisks indicated by the magenta arrow**. The color bar to the right shows the value of the *t*-statistic, using a threshold magnitude of 3. Among the features represented are activation both proximal and distal to the contacts (including contralateral side); activation along known anatomic features such as the left cingulate gyrus; and strong negative activation as seen in the bilateral sensorimotor regions. The stimulation used a 32 s block design with an alternating unipolar 8 mA pulse at 20 Hz. The TR was 2 s, over four blocks for a 5 min acquisition. The patient was under general anesthesia (3).

The major results of the DES-fMRI experiments has been recently reported (3), and was performed safely and successfully in four patients using the methods described above. Figure 4 shows an example from one patient (#5 in Table 1) who was stimulated in the left orbito-frontal region, as shown by the small black asterisk highlighted by the magenta arrow. The overlaid color represents the magnitude of the statistical map (*t*-score) as indicated by the color bar. This image reveals many of the salient features from all patients: (1) robust BOLD activation can be induced; (2) activation is seen both proximally (e.g., adjacent insula and hippocampus) and distally (e.g., opposite hemisphere); (4) the patterns of activation are suggestive of underlying networks, e.g., the strong linear activation along the limbic system of the cingulate gyrus; and (5) robust deactivation (or “negative” activation) is seen, which also appears to conform to underlying networks. We hypothesize for this research that the magnitude of the *t*-score is our third measure of connectivity. Due to the inherent three-dimensional nature of MRI, a complete map of connectivity can be produced and compared with other measures.

### dMRI connectivity

**Figure 5:**
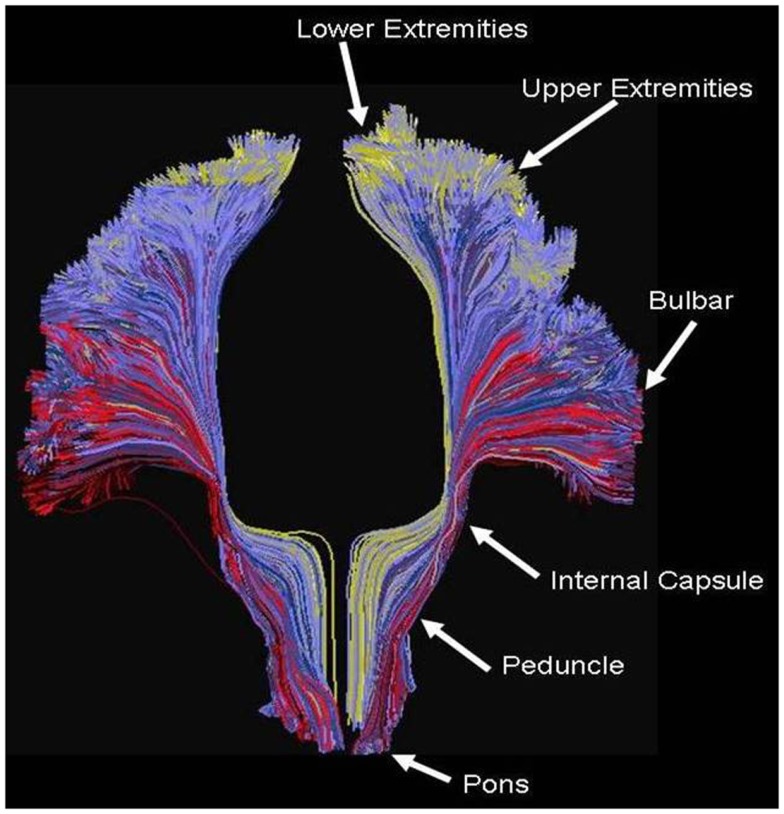
**Example of tractography using the method developed for this work, using as a seed the cortical spinal tract at the level of the central pons, and displaying all tracts connecting that location to all voxels located in the precentral gyrus**. Each path is color-coded by the strength of the global connectivity score as described in the Data Sheet 1 in Supplementary Material (yellow-green represents high structural connectivity; blue is intermediate; red is low). The method recapitulates the known set of pathways connecting both medial and lateral aspects of the precentral gyrus, and is not significantly affected by crossing fibers from the transcallosal and superior longitudinal fasciculus.

Figure 5 shows an example of the PDE method of tractography, using for the seed the mid-pons, and for the target the entire neocortex. A total of 200,000 tracks are produced, but for clarity only the 14,000 connecting to the precentral gyrus are displayed, which is an anterior view showing the resulting cortico-spinal tracks. Each pathway is color-coded by the magnitude of the pathway-score described in the methods. Note the method successfully tracks to all portions of the precentral gyrus, and is not affected by problems from crossing fibers from the corpus callosum or the superior longitudinal fasciculus. As expected, there is relatively strong connectivity to the upper and lower extremities, and lower connectivity to the bulbar region. The collective pathway follows the expected twisting-ribbon geometry of the known cortico-spinal tract.

By applying this method to the invasive patients, using as the seed the location of the stimulating electrodes and using as the target the remaining neocortex, a full cortical map can be produced wherein every cortical voxel obtains a value related to the pathway score. We assume for this research that the magnitude of the pathways-score is our fourth measure of connectivity. Again, due to the inherent three-dimensional nature of MRI, a complete map of connectivity can be produced and compared with other measures.

**Figure 6:**
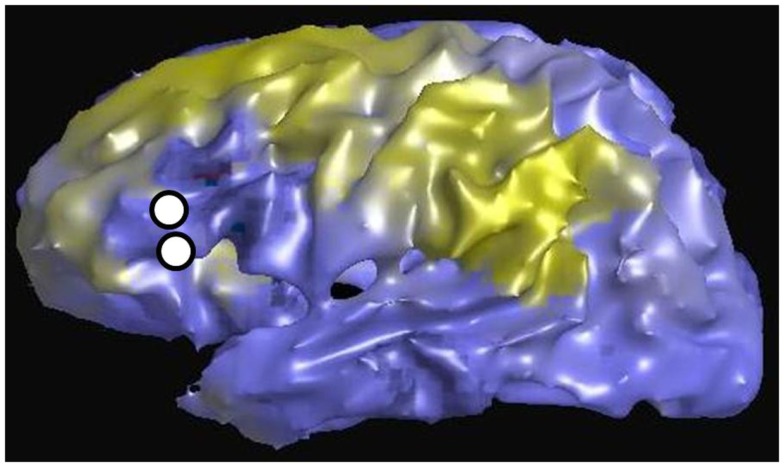
**Left lateral view of structural connectivity, using the electrode locations (white circles) stimulating Broca’s region as a seed and connecting to all other cortical gray matter voxels**. This is the same patient shown in Figures 2 and 3. The green color indicates higher structural connectivity, whereas blue color indicates lower structural connectivity.

Figure 6 is an example of the dMRI method applied to the same patient in Figures 2 and 3, using as a seed the left Broca’s region and then tracking to and scoring all remaining gray matter voxels. The white circles again represent the location of the stimulating electrodes, which were identified to stimulate Broca’s region. The color scale is normalized so that yellow-green is a higher pathway-score than blue. The pattern shows increased connectivity to the presumed language regions of the parietal lobe, in addition to connections in the frontal lobe. This procedure was repeated for all stimulation sites in patients #1–4. Since all cortical voxels can be scored, complete comparisons can be made to the other methods that compute a connectivity value at all cortical voxels, namely rsfMRI and DES-fMRI. CCEP is the only method of the four presented that computes a connectivity score at a relatively small number (100–200) of locations.

### Cross-correlations

**Figure 7:**
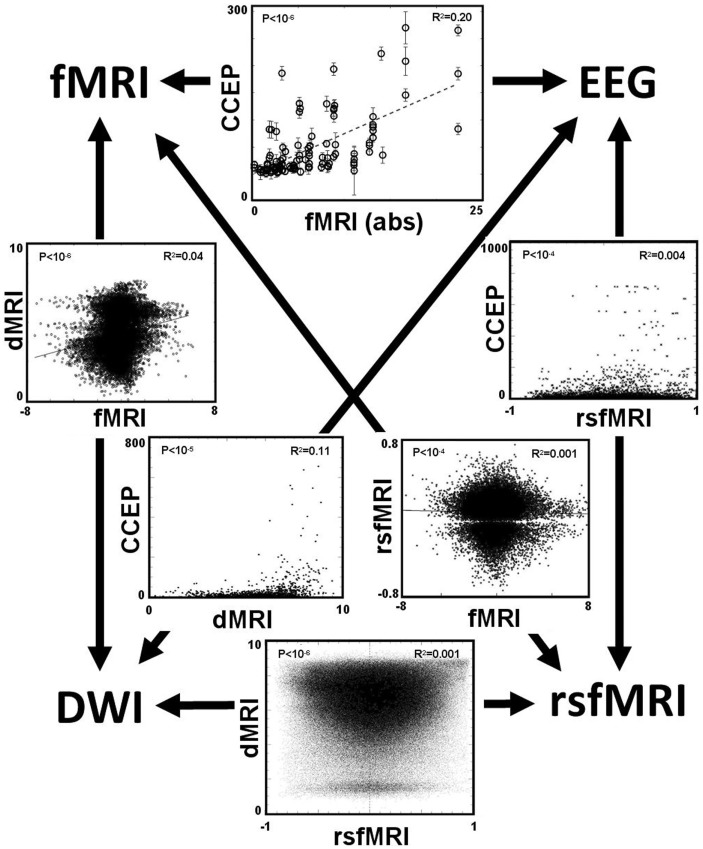
**The main result is displayed as a set of six paired comparisons from four different methods of measuring connectivity**. Each square shows a two-dimensional scatter plot of points using the methods labeled along the axes. The data are a compilation from all eight patients. For data comparing the CCEP method, a point is plotted for each electrode contact. For the other comparisons a point is plotted for each cortical voxel, thus these plots have a higher density of points. The units of the CCEPs scale in μV; the units of the rsfMRI are the Pearson correlation coefficient typically ranging from −1 to 1; the units of fMRI are the statistical *t*-score (using the absolute value when compared with CCEP); and the units of the dMRI are arbitrary with 0 representing negligible structural connectivity and 10 representing strong structural connectivity. The value of the Pearson correlation coefficient *r*^2^ computed from the data within each plot is printed in the top right corner; its associated *p*-value is printed in the top left corner.

After applying these four methods of computing connectivity to a common seed location in an epilepsy patient with intracranial electrodes, a paired comparison can be made between any two selected methods. Figure 7 displays the six different paired comparisons possible from four methods, each shown as a two-dimensional scatter plot with each axis representing the connectivity value of a selected method. The data from different seed locations are superimposed on each plot. In addition, all available data from the eight patients are also superimposed on each plot. For each plot, the Pearson correlation coefficient *r*^2^ and its associated *p*-value are computed from the entire ensemble of displayed data points. These numbers are printed at the top and also listed in Table 3. Hypothesizing that the value of the correlation coefficient is a measure of the consistency between two methods of connectivity, there is a wide range of correspondence ranging from 0.001 to 0.20. All of the correlations show statistical significance, even for the lowest values, and is likely due to the enormous number of data points available for comparison. The lowest correlations are associated with rsfMRI, which the highest are related to CCEPs.

**Table 3:** **Paired comparison of four methods for brain connectivity**.

	CCEP	DES-fMRI	rsfMRI	dMRI
CCEP		*r*^2^ = 0.20	*r*^2^ = 0.004	*r*^2^ = 0.11
		<10^−5^	<10^−4^	<10^−5^
DES-fMRI			*r*^2^ = 0.001	*r*^2^ = 0.04
			<10^−4^	<10^−6^
rsfMRI				*r*^2^ = 0.001
				<10^−6^
dMRI				

*The top number is the Pearson correlation coefficient *r*^2^, and the number below is the associated p-value*.

## Discussion

Although the four measures of connectivity show statistically significant correlation coefficients, the most striking observation for a given pair-correlation is the large scatter of points, particularly on the measures that permit inclusion of all voxels in the brain. That is, for a given pair of measures, there are many pairs of voxels in the brain that show a high level correlation using method A, with a low correlation using method B; and visa versa. While the statistically significant trend is scientifically useful for group comparisons, any possible medical applications to an individual patient require more robust metrics or another paradigm for understanding these discrepancies. There are many possible causes for the low correlation values, some may be intrinsic to the brain and its function, while others are technical and methodological, both of which are now discussed.

One likely contributor to the discrepancy in the pair correlation is the failure to correctly co-localize a point in MRI space with the source of an EEG signal. One reason is technical, that the co-registered position of the electrode as determined by CT is not correctly co-located to the corresponding point on the MRI. The error can be due to either the CT or MRI: brain shift occurring during the presence of implanted electrodes during CT, or warping of the MRI due to field gradients, particularly at the brain’s base. Another reason is the exact location of the voltage source. That is, although the subtraction of an adjacent electrode-contact pair measures a given signal, there exists some uncertainty about the exact location of the source of the signal with respect to the paired contacts. If it is a point source, it is likely that the distance from the source to the electrode pair is comparable to the distance between the electrode contacts. For example, if the contacts are separated by 5 mm, that could represent a distance of two voxels from the imaged locations of the electrodes. This possibility is complicated by the reality that most sources will be distributed, likely over a spatial scale at least as large as 5 mm. One approach to address this consideration is to incorporate a source model of the CCEP waveforms (16) rather than directly use the electric signals that come from the equipment. However, source modeling is complex and introduces its own assumptions and uncertainties.

In addition to robust positive BOLD activation seen during stimulated fMRI, there are network-like regions of “negative” activation, or relative deactivation. This phenomenon is often seen with task-related paradigms and sometimes is attributed to the design, for example where the “rest” cycle is not truly at rest. However, in our DES-fMRI experiments the patient is anesthetized and the negative BOLD patterns appear as a consequence of positive stimulation. It is uncertain if this negative activation is the result of direct point-to-point action potentials from the stimulated region to the negatively activated cortex, or the result of positive stimulation to secondary cortex that in turn deactivates cortex. Regardless, it raises the question of how a shower of signals delivered to cortex results in relative deactivation. One possible explanation is that there is tremendous neuronal processing that occurs in a segment of cortex before electrical responses that synchronize sufficiently to produce a macroscopic voltage capable of detection with intracranial or extracranial electrodes. For example, it is known that an area at least 10 cm^2^ of synchronized cortex is required for detection by a scalp electrode (17). The vast number of neurons required for this ensemble response is likely much more than the number initially stimulated by an incoming wave of action potentials. Thus, between the moment of initial stimulation and macroscopic signal detection there must be a computational buildup with tremendous intra-cortical processing. Although initially stimulated in a positive sense by a relative small number of neurons, this intra-cortical processing could proceed in either increased or decreased tone, that is, either positive or negative reaction.

Another uncertainty likely contributing to the poor correlation regarding comparisons with CCEP signals is the scalar metric derived from the signal and used for comparison. One detail is which time window of the CCEP signal is most appropriate for comparison? For example, regarding comparison with structural connectivity may best compare with the early time course of the signal, perhaps a time scale comparable to the axonal transit time, say between 5 and 15 ms. On the contrary, regarding comparison with resting state connectivity or DES-fMRI, which likely elicit and more steady-state ensemble reaction of brain activity, a better comparison with structural activity might be to average the signal intensity over a much longer period of time, perhaps 0.1–1.0 s.

A source of variability leading to poor correlation may be the manner of electrical stimulation, particularly with the variables of current and frequency. Regarding current, larger currents will stimulate a larger volume of tissue, which may alter the distal patterns of response (18). Experimentally it is difficult to know the optimum current since the current is raised until a desired effect is noticed, whose threshold can vary in different brain regions. Similarly, the 1 Hz stimulation frequency of CCEP may elicit a different network of activation than at a higher – and more physiologic – frequency. These are all experimental values worthy of exploration in future studies.

All data were derived from patients with long-standing and intractable epilepsy, whose brains feature foci of abnormal cortical excitability. These foci likely correspond to the nodes of some network, which raises the possibility of associated abnormal connectivity. This variation could contribute to the scatter seen with an ensemble of paired correlations.

In addition to the large scatter of paired correlations between two given modalities, there is strong variation of the overall correlation between the various pairs of modalities, for example the correlation between CCEP and DES-fMRI is the largest at *r*^2^ = 0.20, while that between DES-fMRI and rsfMRI (and dMRI and rsfMRI) is the weakest at *r*^2^ = 0.001. This difference may reflect the underlying scale of the modality: at one extreme dMRI reflects simple node-to-node connectivity between any two cortical points, whereas rsfMRI connectivity reflects a more “ensemble” of brain activity including the effects of feedback circuits containing multiple nodes. Thus the correlation coefficient may be highest between modalities featuring simple node-to-node connectivity (for example dMRI, and CCEPS derived from early time measurements), and lowest between any modality compared with rsfMRI. The initial expectancy that different measures of connectivity are mutually consistent may be misguided, for example a strong correlation between structural and functional connectivity, and that a pathway to better understanding one measure of connectivity it a detailed analysis of its difference to other measures.

While the *quantitative* comparison of the different connectivity measures is poor, often the qualitative patterns of the maps can seem similar. For example, the lateral surface images in Figures 2, 3, and 6 are from the same patient for the modalities of CCEPs, rsfMRI, and dMRI connectivity, where the seed for each modality is the left Broca’s area. While detailed pair-correlations of voxel-to-voxel scatter plots show the typical finding of a significant but weak correlation, the overall patterns of correlation compare well qualitatively to the eye. This could raise the possibility that coarse features of connectivity are similar, but there are errors in the details.

**Figure 8:**
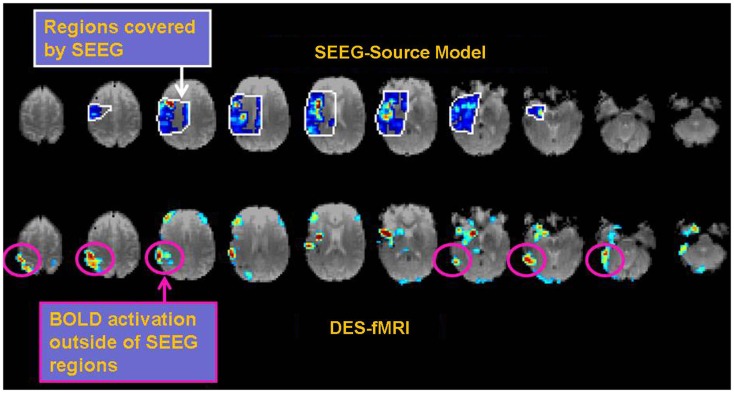
**Example of the possible clinical utility derived from accurate correspondence of different connectivity measures**. While EEG or CCEP measures may form a gold standard, the drawback is limited brain coverage. The use of an EEG source model may improve local coverage between electrodes, but fails to adequately compute electrophysiological activity further outside these regions. The top row of images shows the EEG source model (computed using Brainstorm) from stimulation of an electrode in the right insular region in a patient with 12 right-sided stereotactically placed EEG depth electrodes (not shown). Visualization of the brain’s response to stimulation appears more continuous in the regions of reliable computation (region bounded by white line), and may offer superior comparison to other methods of connectivity. Note the region of reliable results is only covers a minority of the entire brain. The bottom row shows the corresponding DES-fMRI BOLD connectivity map derived from stimulation of the same electrode-pair as the top row. While there is qualitative correspondence of the connectivity maps in the region of the temporal lobe and insula, the DES-fMRI map encompasses the entire brain and reveals strong areas of activation outside the coverage of SEEG electrodes, namely in the right parietal lobe. Establishing a reliable relation between these two measures of connectivity could synergistically enhance the coverage of invasive electrodes.

The practical clinical question arises about how such sophisticated comparisons, metrics, or models could be used to benefit patients with epilepsy. For example, how might knowledge of a network directly help the clinician? The ultimate goal is identification of the EZ, wherein removal of that tissue inhibits the electrophysiological cascade that erupts into a seizure. Assuming the EZ is one node in a network, an alternative approach could be resection of non-EZ node in the network such that its removal interrupts any epileptogenic circuitry that contributes to seizure generation. One of the major problems in the process of the presurgical localization of the EZ through scalp EEG recordings is the issue of false localization of a surface activity that is the result of a network/subcortical spread from a distant focus (in a different gyrus, lobe and at times hemisphere) (19). Optimizing non-invasive measures of connectivities would undoubtedly assist in the identification of the correct focus and would therefore result in the optimization of the surgical results through the resection of the source of the electrical activity rather than a non-needed resection of the wrong falsely localized “focus.” Another approach to how epilepsy might benefit from an accurate relation of the different measures of connectivity is discussed in Figure 8. The data in Figure 8 illustrate the more comprehensive nature of DES-fMRI for the mapping of all the nodes of a particular epileptic network as fMRI measures BOLD changes in the whole brain while depth or subdural electrodes measure a much more restricted part of the cortex that is based on a hypothesis that is generated from less than optimal non-invasive methods as discussed above.

One possible approach is to compare activated networks that are indigenously and spontaneously activated by interictal discharges to those stimulated externally. A technical problem to surmount is that, at a sufficiently small spatial scale, the exact location of depth electrode contacts can be relatively random with respect to the exact location of the EZ. But if sufficiently close, stimulation of the electrode contacts involving the EZ might activate the same spatial network as indigenous activation from an interictal discharge. If these patterns can be observed, for example using the full 3D capability of BOLD imaging, then overlap of the two maps can serve as verification that the location of stimulation by an electrode is the same location as the EZ. In effect, the BOLD pattern revealed by an interictal discharge could serve as a fingerprint regarding the origin of activation (the epileptic focus). In the far future, an enticing strategy would be any new method that can elicit an interictal discharge, or a seizure, which can be turned on and off as desired, and thereby forms the “task” in a BOLD fMRI experiment. This might be accomplished using a pharmacological method to “stress” the system, or modulate the thresholds to uncover epileptic activities in a controlled fashion.

Another approach is to examine the local vs. distal patterns of activation. The hypothesis could be that local cortical activation in the region of the EZ is augmented by the underlying disease and seizure history. Similar to a spreading depression, and propagated by innumerable interneurons along the cortical layers, local activation could appear different than distal stimulation elicited by long range white matter fiber tracks in terms of both amplitude and speed. For example, the mono-synaptic character of long range connections may proceed at a faster rate than polysynaptic connections within the cortical layers. (20) The presence of disease could alter this comparison whereby local activation proceeds abnormally quickly with abnormally high magnitude. The concept of altered local reactivity is supported by electrophysiological observations to local electrodes upon stimulation of the EZ, wherein the magnitude of local electrodes is exaggerated (21).

One potential avenue of failure represented in Figure 7 may result from an ill-posed assumption, namely that the correct comparison between different modalities is a simple pairwise correlation between them. Perhaps a better metric for one modality may incorporate information from other modalities. For example, functional connectivity could be informed from structural connectivity and thereby correct or exclude comparisons that that are not structurally connected. Further, functional connectivity between any pair of points may be influenced more by a multi-nodal network that connects them rather than a single point-to-point connection. This possibility suggests the future importance of a complete brain model that incorporates all the measurable modalities. Such a model can, in effect, translate between the metrics of different modalities. The ultimate goal would be a sufficiently sophisticated model that could conceivably model an individual brain. Such a model could not practically occur at the microscopic spatial scale of neurons, but at the mesoscopic scale of the imaging voxel. The challenge is finding a method to reliably inform the model, i.e., set all the innumerable parameters with information obtained from a non-invasive modality. Such a modality would need to be sufficiently content-rich to inform a large model. One possibility would be long-term resting state fMRI, informed by structural imaging from dMRI methods. This development would represent the next step in the evolution of neuroimaging, in which the imaging biomarker moves from being the images themselves, to a mathematical brain model that is informed by images.

## Conclusion

A significant next step in the future of imaging brain function is connectivity; however, there are many different metrics for connectivity. This work presents experimental observations with cross-comparisons of four methods produced from eight epilepsy patients with intracranial electrodes. The major result is that although the four methods show statistically significant paired-consistency as computed by a non-zero correlation value, the magnitude of the correlations is relatively poor. Thus there is less cross-modal consensus than might be expected with a simple view of brain connectivity. For example, using two modalities A and B, there are many regions of the brain that show strong connectivity using A but low connectivity using B; and visa versa. The reason for the discrepancies is likely inherent to fundamental differences in the different modalities, thus the objective of a strong simple pairwise correlation is ill-posed. However, we envision that strong correlations can be recovered with the use of an intermediary mathematical model of the brain that can translate the connectivity between different modalities.

## Conflict of Interest Statement

The authors declare that the research was conducted in the absence of any commercial or financial relationships that could be construed as a potential conflict of interest.

## Supplementary Material

The Supplementary Material for this article can be found online at http://www.frontiersin.org/Journal/10.3389/fneur.2014.00272/abstract
