# Supplementary material for: Low Consistency of Four Brain Connectivity Measures Derived from Intracranial Electrode Measurements
Source: Front Neurol. 2014 Dec 19;5:272. doi: 10.3389/fneur.2014.00272 (PMC4271609; doi:10.3389/fneur.2014.00272)
Supplement: Supplementary file 1 [file Data_Sheet_1.DOCX]

**Appendix**

The solution of the PDE method of Zhang is a track-density map, however deriving a single trajectory is ill-defined when using the method to connect two points. The resulting track-density map is a scalar field, without direction, forming a pattern of voxels with varying track-counts between a seed and a target. The work of Zhang has now been extended to not only include a track-density at every voxel, but a mean flux vector at every voxel, representing the mean “flow” of paths through that point. This concept is analogous to the Monte Carlo method in which the directions of all paths going through a voxel are recorded. While a useful advance, there is no single unique line that connects two points; rather a set of lines connects them that fills the computational volume. This view is analogous to how a viscous fluid might flow in a basin between a point-source and a point-sink, in which there are infinitely many streamlines connecting them and spread throughout the volume. One possible alternative, which is adopted for this work, is to compute the PDE not between two points, but from a point to an extended boundary, as shown schematically in Fig. A1. Every point in the boundary can then be connected to the seed by a unique trajectory. A useful property of the PDE method is that the resulting manifold of trajectories preserve their topological relationships, and that individual lines generally do not intersect or cross (except at locations with a saddlepoint solution). This construct has direct applications to the clinical procedure of stimulating one intracranial electrode and recording the remaining distal electrodes throughout the brain. At a different level, this approach seems attractive given the mechanisms of embryological development involving axonal path finding, in which a growing axonal cone advances along chemical concentration gradients caused by distal sources, an effect heavily influenced by diffusion processes. Thus, a PDE method based on a diffusion process may be a natural way to describe white matter pathways in the mature brain.

While the method of connecting a point source to a boundary yields a family of lines, some lines may be more important than other lines, perhaps reflecting a varying density of axons along the lines. Therefore, not only is it important to compute the pathway connecting two points, but to assign a score to each line so they can be compared and ranked. There are numerous methods to score a path, and many of them involve the average of some quantity along the path, for example the mean fractional anisotropy (FA), or mean transverse diffusivity. Such average measures have the disadvantage of relative insensitivity to short segments of the path that may have prohibitively low values. One different approach is to score every point along the path with respect to the direction of the path to the underlying Fiber Orientation Distribution (FOD), in effect taking an inner product of a vector pointing parallel to the path to the 2D surface representing the FOD (Raffelt 2012; Dell'Acqua 2013). As schematically shown in Fig A2, if the path is parallel to the major axis of an FOD with an ellipsoid-like shape, there will be a high inner product; if the path is perpendicular to the major axis then there will be a low inner product. If the FOD is spherical, then the inner product is the same regardless of the orientation of the pathway. Given a family of lines throughout a volume of voxels containing FODs, an inner product can be computed at each voxel. Each line is thus associated with a series of inner products along its path, which could lend themselves to the computation of an overall “score” for structural connectivity of that line (see Fig 3A). As one possible example, once these values are computed along the entire path, they can be multiplied together to form a geometric mean. Such a product is suggestive of a probabilistic score, measuring the probability to step from the first point to the second point, times the probability to move from the second point to the third point, and so forth. If the FOD at all voxels were a sphere of unity, such that the inner product of any pathway is unity, then the score for any path will be a large product of numerous ones, which is unity, and this forms a useful measure of the strength of a path. Note that this method raises sensitivity to short segments of the pathways that are orthogonal to a section of strongly oriented fibers, since the inner product at those locations will be low, and the total score of the pathway will be low. For example, if a pathway had 99 points going through a spherical FOD, but one point the inner product is 0.1, then the final score will be 1^99^ * 0.1 = 0.1. An advantage of this method is that it can yield an exponential range of values, permitting the score of a “perfect” pathway as unity, and unlikely pathways as values such as 10^-10^. A consequence of this method, which computes the pathways of all lines from a point to a surface and then scores each line, is that some lines may have scores so low as to be considered “virtual” lines. Such connections have nonzero values of connectivity, but the values are so low as to be, in reality, impossible.

**REFERENCES**

Dell'Acqua F, Simmons A, Williams SC, Catani M., Can spherical deconvolution provide more information than fiber orientations? Hindrance modulated orientational anisotropy, a true-tract specific index to characterize white matter diffusion. Hum Brain Mapp. 2013 Oct;34(10):2464-83

Raffelt D, Tournier JD, Rose S, Ridgway GR, Henderson R, Crozier S, Salvado O, Connelly A. Apparent Fibre Density: a novel measure for the analysis of diffusion-weighted magnetic resonance images. Neuroimage. 2012 Feb 15;59(4):3976-94.
