# Supplementary figures and images for: Low Consistency of Four Brain Connectivity Measures Derived from Intracranial Electrode Measurements
Source: Front Neurol. 2014 Dec 19;5:272. doi: 10.3389/fneur.2014.00272 (PMC4271609; doi:10.3389/fneur.2014.00272)

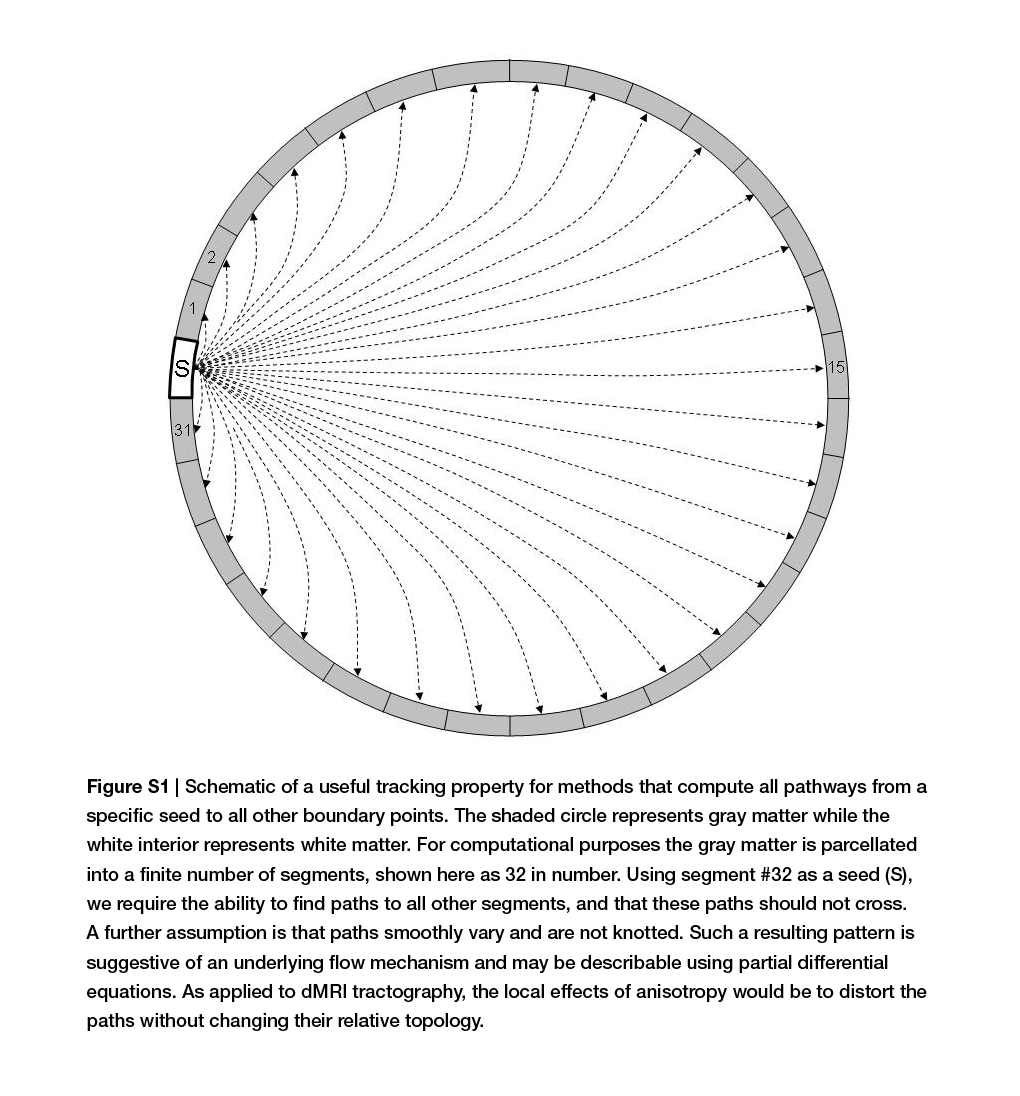

Supplement: Supplementary file 2 [file Image_1.TIF]

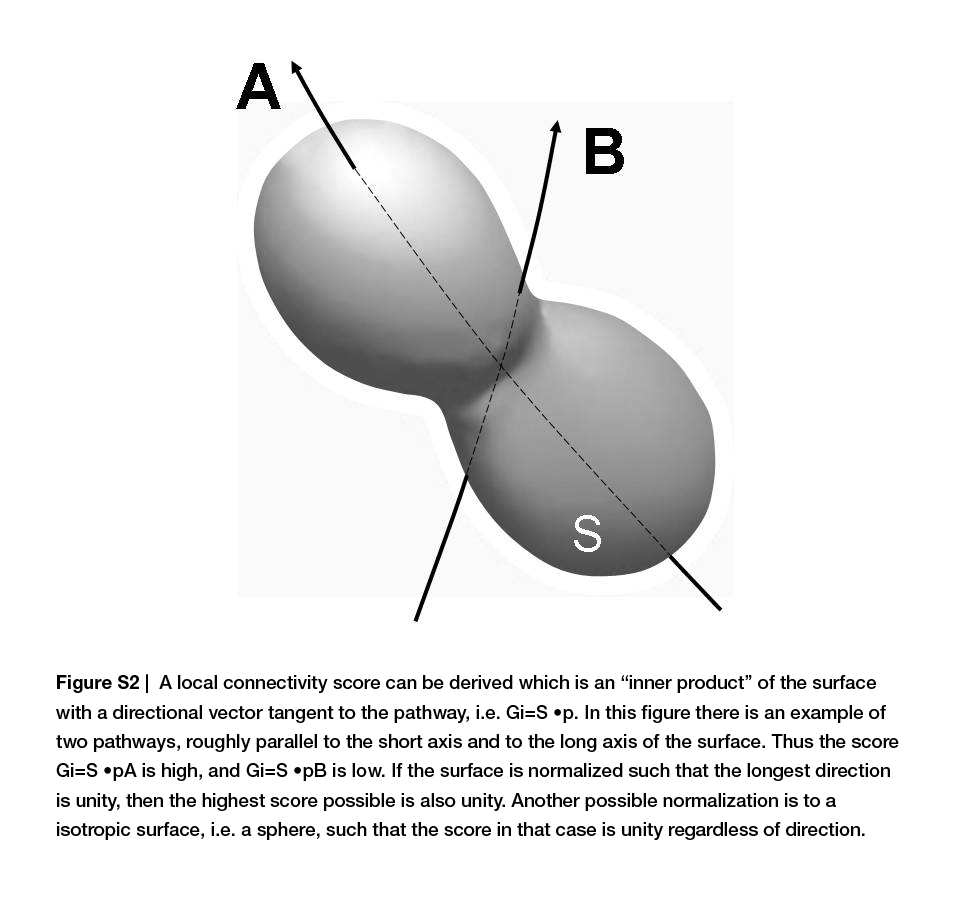

Supplement: Supplementary file 3 [file Image_2.TIF]

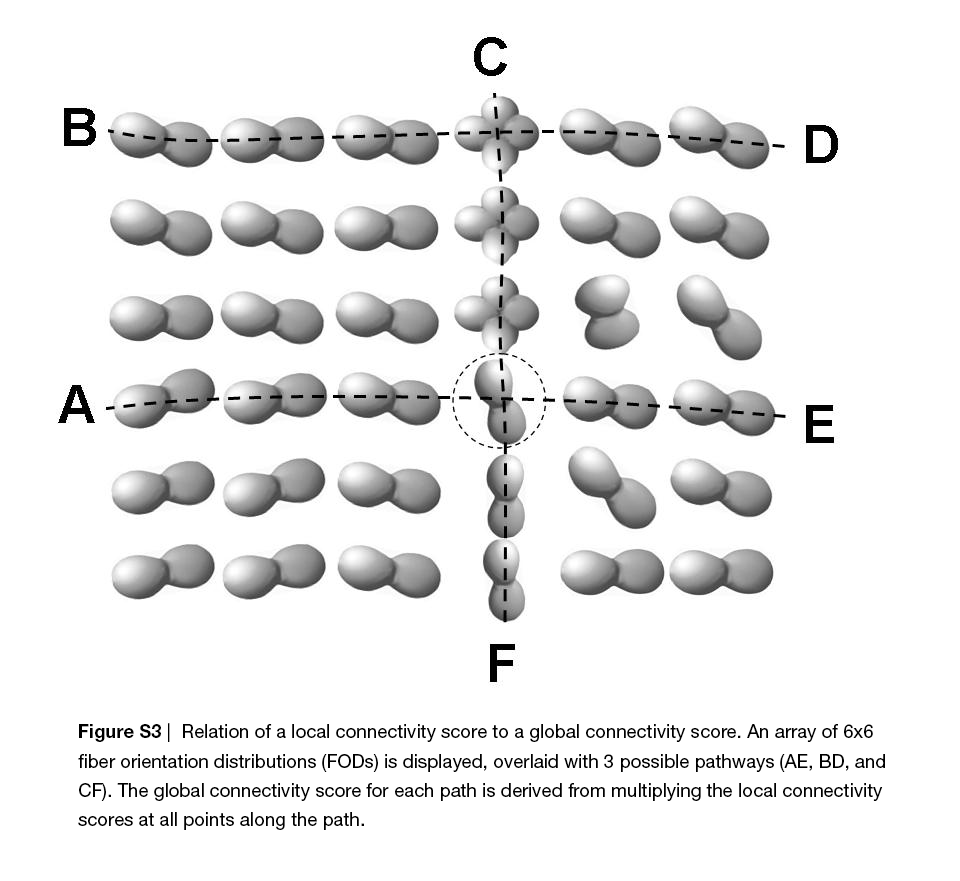

Supplement: Supplementary file 4 [file Image_3.TIF]
